# Perinatal deaths from birth defects in Hunan Province, China, 2010–2020

**DOI:** 10.1186/s12884-023-06092-5

**Published:** 2023-11-13

**Authors:** Xu Zhou, Donghua Xie, Jian He, Yurong Jiang, Junqun Fang, Hua Wang

**Affiliations:** 1 Hunan Provincial Maternal and Child Health Care Hospital, Changsha, Hunan Province China; 2 The Hunan Children’s Hospital, Changsha, Hunan Province China; 3 National Health Commission Key Laboratory of Birth Defects Research, Prevention and Treatment, Hunan Provincial Maternal and Child Health Care Hospital, Changsha, China

**Keywords:** Perinatal deaths, Congenital abnormalities, Risk, Epidemiology, Perinatal mortality

## Abstract

**Objective:**

To describe the perinatal mortality rate (PMR) of birth defects and to define the relationship between birth defects (including a broad range of specific defects) and a broad range of factors.

**Methods:**

Data were obtained from the Birth Defects Surveillance System in Hunan Province, China, 2010–2020. The prevalence rate (PR) of birth defects is the number of birth defects per 1000 fetuses (births and deaths at 28 weeks of gestation and beyond). PMR is the number of perinatal deaths per 100 fetuses. PR and PMR with 95% confidence intervals (CI) were calculated using the log-binomial method. Chi-square trend tests (*χ*^*2*^_*trend*_) were used to determine trends in PR and PMR by year, maternal age, income, education level, parity, and gestational age of termination. Crude odds ratios (ORs) were calculated to examine the association of each maternal characteristic with perinatal deaths attributable to birth defects.

**Results:**

Our study included 1,619,376 fetuses, a total of 30,596 birth defects, and 18,212 perinatal deaths (including 16,561 stillbirths and 1651 early neonatal deaths) were identified. The PR of birth defects was 18.89‰ (95%CI: 18.68–19.11), and the total PMR was 1.12%(95%CI: 1.11–1.14). Birth defects accounted for 42.0% (7657 cases) of perinatal deaths, and the PMR of birth defects was 25.03%. From 2010 to 2020, the PMR of birth defects decreased from 37.03% to 2010 to 21.00% in 2020, showing a downward trend (*χ*^*2*^_*trend*_ = 373.65, *P* < 0.01). Congenital heart defects caused the most perinatal deaths (2264 cases); the PMR was 23.15%. PMR is highest for encephalocele (86.79%). Birth defects accounted for 45.01% (7454 cases) of stillbirths, and 96.16% (7168 cases) were selective termination of pregnancy. Perinatal deaths attributable to birth defects were more common in rural than urban areas (31.65% vs. 18.60%, OR = 2.03, 95% CI: 1.92–2.14) and in females than males (27.92% vs. 22.68%, OR = 1.32, 95% CI: 1.25–1.39). PMR of birth defects showed downward trends with rising maternal age (*χ*^*2*^_*trend*_ = 200.86, *P* < 0.01), income (*χ*^*2*^_*trend*_ = 54.39, *P* < 0.01), maternal education level (*χ*^*2*^_*trend*_ = 405.66, *P* < 0.01), parity (*χ*^*2*^_*trend*_ = 85.11, *P* < 0.01) and gestational age of termination (*χ*^*2*^_*trend*_ = 15297.28, *P* < 0.01).

**Conclusion:**

In summary, birth defects are an important cause of perinatal deaths. Rural areas, female fetuses, mothers with low maternal age, low income, low education level, low parity, and low gestational age of termination were risk factors for perinatal deaths attributable to birth defects. Future studies should examine the mechanisms. Our study is helpful for intervention programs to reduce the PMR of birth defects.

## Introduction

Perinatal deaths were the combination of fetal and neonatal deaths, including infant deaths that occur at less than 7 days of age and fetal deaths with a stated or presumed period of gestation of 28 weeks or more [1]. Birth defects are structural or functional anomalies at or before birth [2]. The accepted prevalence rate (PR) of birth defects is about 2–3% worldwide [3]. Severe birth defects significantly increase the perinatal death risk [4–7]. In developed countries such as Europe and the US, birth defects have been the leading cause of perinatal death and infant death for a long time [8]. WHO estimated that about 12.6% of neonatal deaths worldwide each year are related to birth defects [9], and 240,000 newborns worldwide die each year from birth defects within the first 28 days of life, and birth defects cause 170,000 children deaths between the ages of 1 month and 5 years [10]. Therefore, the study on birth defects is significant and deserves more attention.

Analysis of the epidemiology of perinatal deaths attributable to birth defects has significant implications for clinical care and consultation [11, 12] and may contribute to reducing perinatal mortality rate (PMR) [13]. There were some studies on perinatal deaths attributable to birth defects. E.g., Heinke et al. have studied the risk of stillbirth for fetuses with specific defects [14]. Andrew et al. found the PR of intrauterine fetal death in gastroschisis was 4.48% [15]. Deng et al. found that the PMR of omphalocele was 52.4% [16]. In Hunan Province, China, 24.96% of cleft lip and palate were perinatal deaths [17]. Helen et al. found that about 3.6% of nonchromosomal congenital heart defects were perinatal deaths [18]. The PMR of birth defects was about 20% in Europe (2003–2007) [19]. Li et al. found that birth defects accounted for 22.45% of perinatal deaths in China (1990–2001) [20]. In Victoria, Australia, birth defects have accounted for approximately 25% of all perinatal deaths [21]. In 1999, birth defects accounted for nearly 1 in 5 infant deaths in the US [22]. However, there are limitations in these studies. First, systematic epidemiological studies on perinatal deaths attributable to birth defects (including a broad range of specific defects) are rare. Second, some studies were limited in data, such as relatively few cases included or surveys conducted in unrepresentative districts or hospitals. There are fewer relevant studies in China. Third, some studies needed to be updated.

Therefore, we conducted an epidemiological study on perinatal deaths attributable to birth defects based on data from the Birth Defects Surveillance System in Hunan Province, south-central China (from 2010 to 2020), to describe the PMR of birth defects, and to define the relationship between birth defects (including a broad range of specific defects) and a broad range of factors.

## Methods

### Data sources

This study is a record analysis of hospital-based surveillance. This study used data from the Birth Defects Surveillance System in Hunan Province, China, 2010–2020, which is run by the Hunan Provincial Health Commission and involves 52 representative registered hospitals in Hunan Province. Surveillance data of fetuses (births and deaths at 28 weeks of gestation and beyond) and all birth defects included demographic characteristics such as residence, gender, maternal age, and other key information.

According to the WHO International Classification of Diseases (Tenth Revision, ICD-10), birth defects were classified into 23 subtypes: anencephaly (Q00), spina bifida (Q05), encephalocele (Q01), hydrocephalus (Q03), cleft palate (Q35), cleft lip (Q36), cleft palate with cleft lip (Q37), microtia/anotia (Q17.2, Q16.0), other external ear defects (Q17), esophageal atresia (Q39), atresia of rectum and anus (Q42), hypospadias (Q54), extrophy bladder (Q64.1), talipes equinovarus (Q66.0), polydactyly (Q69), syndactyly (Q70), limb reduction (Q71, Q72), diaphragmatic hernia (Q79.0), omphalocele (Q79.2), gastroschisis (Q79.3), conjoined twins (Q89.4), Down syndrome (Q90), congenital heart defects (Q20-26) or ‘other’ (Q00-Q99, excluding the codes mentioned above).

### Definitions

Perinatal deaths include stillbirths (fetal deaths with a gestation of 28 weeks or more) and early neonatal deaths (infant deaths less than 7 days of age). PR of birth defects is the number of birth defects per 1000 fetuses (births and deaths at 28 weeks of gestation and beyond). PMR is the number of perinatal deaths per 100 fetuses.

### Ethics approval and consent to participate

The study was approved by the Hunan Provincial Maternal and Child Health Care Hospital. (NO: 2022-S051). It is a retrospective study of medical records; all data were fully anonymized before we accessed them. Moreover, we deidentified the patient records before analysis. We confirmed that all experiments were performed following relevant guidelines and regulations. We confirmed that informed consent was obtained from all subjects and/or their legal guardian(s). Doctors obtain consent from pregnant women before collecting surveillance data, witnessed by their families and the heads of the obstetrics or neonatal departments. Doctors obtain consent from their parents or guardians for live births witnessed by their families and the heads of the obstetrics or neonatal departments. Since the Health Commission of Hunan Province collects those data, and the government has emphasized the privacy policy in the “Maternal and Child Health Monitoring Manual in Hunan Province”, there is no additional written informed consent.

### Data quality control

To carry out surveillance, the Hunan Provincial Health Commission formulated the “Maternal and Child Health Monitoring Manual in Hunan Province”. Data were collected and reported by experienced doctors. To reduce the integrity and information error rates of surveillance data, the Hunan Provincial Health Commission asked the technical guidance departments to conduct comprehensive quality control each year.

### Statistical analysis

We calculated the PR, PMR, and its 95% confidence intervals (CI) by the log-binomial method. Chi-square trend tests (*χ*^*2*^_*trend*_) were used to determine trends in PR and PMR by year, maternal age, income, education level, parity, and gestational age of termination. Crude odds ratios (ORs) were calculated to examine the association of each maternal characteristic with perinatal deaths attributable to birth defects. Statistical analyses were performed using SPSS 18.0 (IBM Corp., NY, USA).

## Results

### Prevalence of total birth defects and perinatal deaths

Our study included 1,619,376 fetuses, a total of 30,596 birth defects in the perinatal period, and 18,212 perinatal deaths (including 16,561 stillbirths and 1651 early neonatal deaths) were identified. The PR of birth defects was 18.89‰(95%CI: 18.68–19.11), and the total PMR was 1.12%(95%CI: 1.11–1.14). Birth defects accounted for 42.0% (7657 cases) of perinatal deaths, and the PMR of birth defects was 25.03%. From 2010 to 2020, the PMR of birth defects decreased from 37.03% to 2010 to 21.00% in 2020, showing a downward trend (*χ*^*2*^_*trend*_ = 373.65, *P* < 0.01), while the PMR attributable to birth defects was stable (Table 1).

**Table 1:** Prevalence of total birth defects and perinatal deaths in Hunan Province, China, 2010–2020

Year	Total fetuses (n)	Total birth defects (n)	Prevalence (‰, 95%CI)	Total perinatal deaths (n)	PMR (%, 95%CI)	Perinatal deaths attributable to birth defects (n)	PMR of birth defects (%)	PMR attributable to birth defects (%)
2010	98,624	1847	18.73(17.87–19.58)	1667	1.69(1.61–1.77)	684	37.03	41.03
2011	107,500	2449	22.78(21.88–23.68)	1690	1.57(1.50–1.65)	749	30.58	44.32
2012	125,583	2574	20.50(19.70-21.29)	1782	1.42(1.35–1.48)	814	31.62	45.68
2013	135,645	2572	18.96(18.23–19.69)	1850	1.36(1.30–1.43)	824	32.04	44.54
2014	143,640	3187	22.19(21.42–22.96)	1932	1.35(1.29–1.41)	818	25.67	42.34
2015	160,629	3508	21.84(21.12–22.56)	1846	1.15(1.10–1.20)	719	20.50	38.95
2016	170,688	3107	18.20(17.56–18.84)	1901	1.11(1.06–1.16)	744	23.95	39.14
2017	196,316	3533	18.00(17.40-18.59)	1694	0.86(0.82–0.90)	686	19.42	40.50
2018	177,762	2900	16.31(15.72–16.91)	1435	0.81(0.77–0.85)	607	20.93	42.30
2019	164,840	2643	16.03(15.42–16.65)	1293	0.78(0.74–0.83)	534	20.20	41.30
2020	138,149	2276	16.47(15.80-17.15)	1122	0.81(0.76–0.86)	478	21.00	42.60
Total	1,619,376	30,596	18.89(18.68–19.11)	18,212	1.12(1.11–1.14)	7657	25.03	42.04

*Abbreviation*: *CI* confidence intervals, *PMR* perinatal mortality rate

### Prevalence of specific defects and perinatal deaths

Table 2 shows perinatal deaths attributable to specific defects, sorted by number of perinatal deaths. Congenital heart defects caused the most perinatal deaths (2264 cases); the PMR was 23.15%. PMR is highest for encephalocele (86.79%), followed by hydrocephalus (84.25%), anencephaly (82.86%), conjoined twins (80.00%), gastroschisis (65.05%), spina bifida (63.64%), diaphragmatic hernia (60.49%), down syndrome (59.09%), and cleft palate with cleft lip (52.12%) (Table 2).

**Table 2:** Prevalence of specific defects and perinatal deaths

Type of birth defect	Specific defects (n)	Prevalence (‰, 95%CI)	Perinatal deaths (n)	PMR of specific defects (%)
Congenital heart defects	9779	6.04(5.92–6.16)	2264	23.15
Hydrocephalus	565	0.35(0.32–0.38)	476	84.25
Cleft palate with cleft lip	777	0.48(0.45–0.51)	405	52.12
Limb reduction	508	0.31(0.29–0.34)	182	35.83
Talipes equinovarus	1226	0.76(0.71–0.80)	155	12.64
Down syndrome	242	0.15(0.13–0.17)	143	59.09
Spina bifida	187	0.12(0.10–0.13)	119	63.64
Diaphragmatic hernia	162	0.10(0.08–0.12)	98	60.49
Cleft lip	447	0.28(0.25–0.30)	97	21.70
Hypospadias	865	0.53(0.50–0.57)	73	8.44
Omphalocele	149	0.09(0.08–0.11)	70	46.98
Gastroschisis	103	0.06(0.05–0.08)	67	65.05
Anencephaly	70	0.04(0.03–0.05)	58	82.86
Atresia of rectum and anus	440	0.27(0.25–0.30)	56	12.73
Encephalocele	53	0.03(0.02–0.04)	46	86.79
Polydactyly	3185	1.97(1.90–2.04)	40	1.26
Esophageal atresia	108	0.07(0.05–0.08)	35	32.41
Other external ear defects	2367	1.46(1.40–1.52)	31	1.31
Syndactyly	1040	0.64(0.60–0.68)	17	1.63
Cleft palate	446	0.28(0.25–0.30)	17	3.81
Microtia/anotia	372	0.23(0.21–0.25)	13	3.49
Conjoined twins	5	0.00(0.00-0.01)	4	80.00
Extrophy bladder	24	0.01(0.01–0.02)	2	8.33

*Abbreviation*: *CI* confidence intervals, *PMR* perinatal mortality rate

### Selective termination of pregnancy (TOP) for stillbirths attributable to birth defects

A total of 16,561 stillbirths were identified, accounting for 90.93% of total perinatal deaths. Birth defects accounted for 45.01% (7454 cases) of stillbirths, and 96.16% (7168 cases) were selective TOP. In other words, most perinatal deaths caused by birth defects were TOP. Similar to the total perinatal deaths, from 2010 to 2020, the stillbirth rate attributable to birth defects was stable (Table 3).

**Table 3:** Selective termination of pregnancy for stillbirths attributable to birth defects

Year	Total stillbirths (n)	Stillbirths attributable to birth defects (n)	Stillbirth rate attributable to birth defects (%)	Selective termination of pregnancy (n)	Proportion in stillbirths attributable to birth defects (%)
2010	1451	643	44.31	606	94.25
2011	1500	724	48.27	698	96.41
2012	1598	791	49.50	755	95.45
2013	1668	798	47.84	770	96.49
2014	1770	789	44.58	759	96.20
2015	1700	700	41.18	678	96.86
2016	1662	744	44.77	694	93.28
2017	1591	686	43.12	655	95.48
2018	1337	607	45.40	586	96.54
2019	1222	510	41.73	510	100.00
2020	1062	462	43.50	457	98.92
Total	16,561	7454	45.01	7168	96.16

### Epidemiology of perinatal deaths attributable to birth defects

Perinatal deaths attributable to birth defects were more common in rural than urban areas (31.65% vs. 18.60%, OR = 2.03, 95% CI: 1.92–2.14) and in females than males (27.92% vs. 22.68%, OR = 1.32, 95% CI: 1.25–1.39). PMR of birth defects showed downward trends with rising maternal age (*χ*^*2*^_*trend*_ = 200.86, *P* < 0.01), income (*χ*^*2*^_*trend*_ = 54.39, *P* < 0.01), maternal education level (*χ*^*2*^_*trend*_ = 405.66, *P* < 0.01), parity (*χ*^*2*^_*trend*_ = 85.11, *P* < 0.01) and gestational age of termination (*χ*^*2*^_*trend*_ = 15297.28, *P* < 0.01) (Table 4).

**Table 4:** Epidemiology of perinatal deaths attributable to birth defects

Characteristics	Birth defects (n)	Perinatal deaths (n)	PMR of birth defects (%)	OR (95%CI)
Region				
Emphasis>Urban	15,530	2889	18.60	**Reference**
Rural	15,066	4768	31.65	2.03(1.92–2.14)
Gender				
Male	18,039	4092	22.68	**Reference**
Female	12,423	3468	27.92	1.32(1.25–1.39)
Unknown	134	97	72.39	
Maternal age (years old)				
< 20	546	203	37.18	1.96(1.64–2.34)
20–24	6241	2068	33.14	1.64(1.53–1.75)
25–29	13,018	3022	23.21	**Reference**
30–34	7186	1521	21.17	0.89(0.83–0.95)
≥ 35	3605	843	23.38	1.01(0.93–1.10)
Per-capita annual income (¥)				
< 2000	928	329	35.45	1.75(1.52–2.01)
2000-	4448	1198	26.93	1.17(1.09–1.26)
4000-	8387	2106	25.11	1.07(1.00-1.13)
8000-	16,833	4024	23.91	**Reference**
Maternal education level				
Illiteracy or primary school	622	244	39.23	1.79(1.52–2.11)
Secondary school	7277	2234	30.70	1.23(1.15–1.31)
Senior school	11,414	3024	26.49	**Reference**
University or above	11,283	2155	19.10	0.66(0.62–0.70)
Parity history				
No	272	209	76.84	9.60(7.23–12.75)
Once	17,869	4589	25.68	**Reference**
Tiwce	10,856	2485	22.89	0.86(0.81–0.91)
≥ 3 times	1599	374	23.39	0.88(0.78-1.00)
Gestational age of termination				
28–33 weeks	6099	5070	83.13	87.35(79.77–95.64)
34–36 weeks	4196	1503	35.82	9.89(9.06–10.80)
≥ 37 weeks	20,301	1084	5.34	**Reference**

*Abbreviation*: *PMR* perinatal mortality rate, *OR* crude odds ratio, *CI* confidence intervals

## Discussion

Overall, birth defects are an important cause of perinatal deaths. Our study is the most recent systematic study on perinatal deaths attributable to birth defects in China. Our discovery makes a significant original contribution to the field.

There were several meaningful findings in this study. First, from 2010 to 2020, the PMR of birth defects showed a downward trend, while the PMR attributable to birth defects was stable. The PMR of birth defects was 25.03% in this study, higher than in some high-income countries (about 20% in Europe) [19]. From 2010 to 2020, the PMR of birth defects showed a downward trend, which may be mainly related to economic and medical conditions, as advanced therapeutic tools and better economic conditions are good for the survival of children with birth defects [23]. The downward trend indicated that the economic and medical conditions in Hunan Province were improving. In addition, improvements in prenatal screening and diagnosis led to many severe birth defects being terminated before 28 weeks of gestation [24, 25].

In our study, birth defects accounted for 42.0% of perinatal deaths, and the PMR attributable to birth defects was stable in 2010–2020. It seems higher than in some high-income countries (about 20-30%) [21, 22] and also higher than previous studies in China (22.45% in China, 1990–2001) [20]. In most national reports, birth defects account for less than 10% of all stillbirths after 22 weeks of gestation, with a median of 7.4% [26]. Since the major risk factors (mainly non-communicable disorders) for stillbirth in China are similar to those in high-income countries [7, 26, 27], and most (96.16%) stillbirths attributable to birth defects in our study were selective TOP, we infer that the main reason for the high PMR attributable to birth defects was selective TOP. In addition, many birth defects may be combined with other serious diseases, and perinatal deaths are partly caused by birth defects. Aldo et al. found that infant mortality rates attributable to birth defects significantly decreased from 1950 to 1994 in Western Europe, North America, and Oceania, while in some areas (such as Spain, Portugal, Mexico, and Eastern Europe) remained stable or increased. It has been suggested that in Portugal, such a trend may partly be attributable to improved diagnosis and case notification associated with an increase in hospital-based deliveries [8]. The reason for the PMR attributable to birth defects was stable in 2010–2020, which may be similar to Aldo’s study.

Second, there were significant differences in the PMR of specific defects. Usually, specific defects with high PMR were easy to diagnose and significantly impacted the patients (primarily physiological functions, secondarily appearance malformations). In this study, the most common specific defects that impact the physiological functions with high PMR include hydrocephalus, Down syndrome, spina bifida, diaphragmatic hernia, gastroschisis, anencephaly, encephalocele, and conjoined twins, and the most common specific defects that impact the with high PMR include cleft palate with cleft lip. It is similar to previous studies [14]. However, the PMR (or stillbirth rate) of specific defects varied dramatically across studies [14–16, 18, 28]. E.g., Dominique et al. found that the stillbirth rate ranged from 11‰ with bladder exstrophy to 490‰ with limb-body-wall complex [14]. It may be mainly related to prenatal diagnosis and selective TOP [29–32]. In addition, differences in study methods (such as study period and pregnancy outcome indicators) make it difficult to compare results across studies. The important contribution of this study is the systematic analysis of perinatal deaths for a range of defects, which is informative.

Third, some factors increase the risk of perinatal deaths attributable to birth defects, including rural areas, female fetuses, mothers with low maternal age, low income, low education level, low parity, and low gestational age of termination. There were several interesting findings. (1) Previous studies have shown birth defects were more common in males than females [33–35], including some severe birth defects (such as hypospadias, severe congenital heart defects, and congenital anal atresia) [36–38]. However, in our study, the PMR of birth defects was higher in females than males. The possible explanation for this phenomenon may be the higher prenatal diagnosis rate of birth defects for females than for males and the higher TOP rate in females [39]. In addition, the “boy preference” phenomenon exists in some areas of China, especially in poor rural areas, which may also increase the PMR of birth defects for females [40]. (2) Rural areas, mothers with low income, and low education levels all reflect low economic conditions. As mentioned before, low economic conditions increase the risk of perinatal deaths. Several previous studies also supported this conclusion [41–45]. (3) Low maternal age and low parity are similar indicators, reflecting mainly high reproductive ability and partly low economic conditions, encouraging mothers to terminate fetuses with birth defects and try to conceive healthy babies. In addition, low maternal age was associated with severe specific defects, such as gastroschisis and cleft lip and palate [46, 47], which increased the PMR. (4) Low gestational age of termination is associated with improved prenatal screening and diagnosis. As most perinatal deaths attributable to birth defects were selective TOP, the earlier birth defects are terminated, the less impact they will have on pregnant women [48, 49]. More and more birth defects are terminated before 28 weeks of gestation, while in our study, birth defects before 28 weeks of gestation were not included.

Some things could be improved in our study. First, our study only includes birth defects at 28 weeks of gestation and above due to data limitations. A considerable proportion of birth defects are diagnosed and terminated before 28 weeks of gestation (such as Down syndrome), significantly impacting the death rate of birth defects. Second, as mentioned before, many factors are correlated, and a multifactorial analysis of risk factors of perinatal deaths attributable to birth defects would be appropriate. However, we could not conduct a multifactorial analysis due to data limitations, as the Birth Defects Surveillance System in Hunan Province was electronically rebuilt in 2012 and 2015, respectively, and some key information could not be effectively combined. Third, multiple birth defects may increase the risk of perinatal death. In our study, many birth defects were multiple birth defects. However, we did not analyze it. Fourth, there are large differences in the severity of some subtypes of specific defects with high PR, such as congenital heart defects. Further analyses of perinatal deaths in the different subtypes of those specific defects would be appropriate.

## Conclusion

In summary, birth defects are an important cause of perinatal deaths. Rural areas, female fetuses, mothers with low maternal age, low income, low education level, low parity, and low gestational age of termination were risk factors for perinatal deaths attributable to birth defects. Future studies should examine the mechanisms. Our study is helpful for intervention programs to reduce the PMR of birth defects.

## Data Availability

All data generated or analyzed during this study are included in this published article.
